# Evolution of a website: CAHPepTalk.com & the development of the emergency hydrocortisone mobile app

**DOI:** 10.1186/1687-9856-2015-S1-P54

**Published:** 2015-04-28

**Authors:** Irene Mitchelhill, Nat Jackson, Jennie King, Dalia Saad

**Affiliations:** 1 Sydney Children’s Hospital (SCHN), Randwick, NSW, Australia; 2 Jacksonspeed.com: Film Editing & Web Design, Pacific Highway, St Leonard’s NSW Australia; 3 Sydney Nursing School, University of Sydney, Sydney, NSW, Australia; 4 Pfizer Australia, West Ryde NSW Australia

## 

A comprehensive, validated psychosocial education program (PEP) titled “The CAH Family Workshop” was developed a decade ago to meet the needs of families with Congenital Adrenal Hyperplasia (CAH). Engaging professionals experienced in the field of endocrinology, together with information from parent interviews, was key to development of the program’s content. Validation of the program was conducted with the families participating in the program, from one of the three tertiary Children’s Hospitals in NSW Australia. The PEP provides families with essential information to assist in understanding and managing the condition.

An awareness of a lack of CAH resources for families living in rural and remote regions of NSW and Australia led to the development of the program into a DVD format. This enabled these families access to the program which covers all aspects of care. The DVD format was designed to be facilitated by one experienced health professional thus reducing the need for a team of specialists to travel to country areas, which was logistically difficult and costly.

The need for CAH resources was further identified in the neighbouring South-East Asian countries of Vietnam and Indonesia where there is a higher incidence of CAH. Through the support of volunteer medical professionals and health services interpreters, the program was translated into these languages. The provision of this valuable resource for families and health professionals in these countries was supported by Caring Living As Neighbours (CLAN) and the CAH Support Group of Australia (CAHSGA).

The most recent stage of this project is the development of the CAHPepTalk.com website, which provides easy access to the English, Vietnamese and Indonesian translations of the program and relevant resources. This presentation will discuss the process of developing this patient-focused education website together with a new Hydrocortisone Emergency Injection App for iphones & android devices.

